# Redox homeostasis and age‐related deficits in neuromuscular integrity and function

**DOI:** 10.1002/jcsm.12223

**Published:** 2017-07-26

**Authors:** Giorgos K. Sakellariou, Adam P. Lightfoot, Kate E. Earl, Martin Stofanko, Brian McDonagh

**Affiliations:** ^1^ GeneFirst Ltd Culham Science Centre Abingdon Oxfordshire OX14 3DB UK; ^2^ School of Healthcare Science Manchester Metropolitan University Manchester M1 5GD UK; ^3^ MRC‐Arthritis Research UK Centre for Integrated Research into Musculoskeletal Ageing, Department of Musculoskeletal Biology, Institute of Ageing and Chronic Disease University of Liverpool Liverpool L7 8TX UK; ^4^ Microvisk Technologies Ltd The Quorum 7600 Oxford Business Park Oxford OX4 2JZ UK; ^5^ Department of Physiology, School of Medicine National University of Ireland Galway Ireland

**Keywords:** Frailty, Superoxide dismutase, Neuromuscular junction, Mitochondria, Redox signalling, Motor neurons

## Abstract

Skeletal muscle is a major site of metabolic activity and is the most abundant tissue in the human body. Age‐related muscle atrophy (sarcopenia) and weakness, characterized by progressive loss of lean muscle mass and function, is a major contributor to morbidity and has a profound effect on the quality of life of older people. With a continuously growing older population (estimated 2 billion of people aged >60 by 2050), demand for medical and social care due to functional deficits, associated with neuromuscular ageing, will inevitably increase. Despite the importance of this ‘epidemic’ problem, the primary biochemical and molecular mechanisms underlying age‐related deficits in neuromuscular integrity and function have not been fully determined. Skeletal muscle generates reactive oxygen and nitrogen species (RONS) from a variety of subcellular sources, and age‐associated oxidative damage has been suggested to be a major factor contributing to the initiation and progression of muscle atrophy inherent with ageing. RONS can modulate a variety of intracellular signal transduction processes, and disruption of these events over time due to altered redox control has been proposed as an underlying mechanism of ageing. The role of oxidants in ageing has been extensively examined in different model organisms that have undergone genetic manipulations with inconsistent findings. Transgenic and knockout rodent studies have provided insight into the function of RONS regulatory systems in neuromuscular ageing. This review summarizes almost 30 years of research in the field of redox homeostasis and muscle ageing, providing a detailed discussion of the experimental approaches that have been undertaken in murine models to examine the role of redox regulation in age‐related muscle atrophy and weakness.

## Introduction

**Figure 1:**
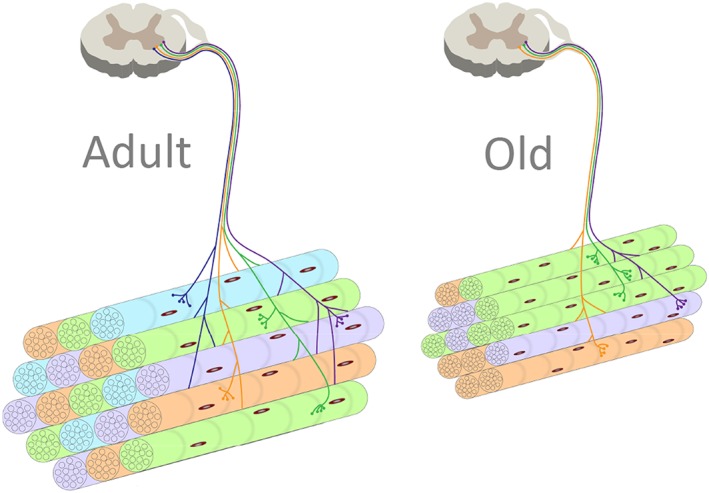
Schematic representation of the morphological neuromuscular alterations/impairments that occur with the advance of age. Ageing skeletal muscle is associated with increased fibre‐type grouping due to continual cycles of denervation and reinnervation. Axonal degeneration and motor neuron death, inherent with aging, leads to reduced number of motor axons innervating myofibres. These events inevitably result in loss of motor units and atrophy of the remaining muscle cells.

Ageing is characterized as the time‐dependent functional decline of cells, organs and tissues throughout the body^1^ and is the primary risk factor for major human pathologies, including cancer, diabetes, cardiovascular disorders and neurodegenerative/neuromuscular diseases. Loss of skeletal muscle mass and force inherent with ageing has a profound effect on the quality of life of older people. Human investigations have shown that by the age of 70, there is a 25–30% reduction in the cross‐sectional area (CSA) of skeletal muscle and a decline in muscle strength by 30–40%,^2^ associated with neurological impairments including loss of motor units,^3^, ^4^ neuromuscular junction (NMJ) instability,^5^ a decline in motor nerve function^6^ and increased fibre‐type grouping due to continual cycles of denervation and reinnervation^7^ (Figure 1). The reduction in muscle strength with age is associated with an increased mortality risk,^8^ an increased susceptibility to risk of falls and, subsequently, an increased need for residential care. According to the World Health Organization, the global population of elderly people aged >60 years was 600 million in 2000 and is expected to rise to around 2 billion by 2050^9^; thus, increased demand for medical and social care will inevitably increase, rising the financial costs of healthcare systems.^10^ Physical activity can undoubtedly delay the progression of ageing muscle affects,^11^, ^12^ but even physically active older individuals experience age‐associated muscle atrophy and weakness.^13^ Age‐dependent myofibre atrophy is a life‐long process with a complex and multifactorial aetiology that involves both intrinsic and extrinsic factors^7^; despite the importance of this area, elucidation of the primary biochemical and molecular mechanisms underlying the prominent age‐associated decline in muscle mass and function has proven to be difficult.

Oxidative damage has been suggested to be among the factors involved in the loss of tissue function that occurs during ageing, and experimental evidence in humans^14^, ^15^, ^16^ and rodents^17^, ^18^, ^19^ has shown that skeletal muscle exhibits age‐dependent increases in the products of oxidative damage to biomolecules including proteins, lipids and nucleic acids. Recent reports have attributed the positive correlation between age and oxidative damage to age‐related changes in reactive oxygen and nitrogen species (RONS), with skeletal muscle fibres from old rodents exhibiting elevated intracellular RONS levels compared with young/adult rodents.^20^, ^21^ The hypothesis that an increased generation of oxidants *in vivo* plays a key role in age‐related deficits in muscle mass and function has been examined in several transgenic and knockout studies with inconsistent results.

Many reports^19^, ^22^, ^23^, ^24^, ^25^, ^26^, ^27^, ^28^, ^29^, ^30^, ^31^, ^32^, ^33^, ^34^, ^35^ have examined skeletal muscle of rodents lacking and/or overexpressing various key regulatory enzyme systems (in homo/heterozygotic and tissue‐specific models) involved in the reduction and/or generation of oxidants to determine whether specific defects in antioxidant protection and the resultant changes in redox homeostasis influence the onset and/or rate of age‐related muscle atrophy and functional deficits.

Although the skeletal muscle fibre‐type profile differs between murine and human skeletal muscle (myosin heavy chain isoform IIB is not expressed in humans),^36^ neuromuscular ageing in both humans and rodents share similar features. These include, but are not limited to, loss of muscle fibres^37^ and reduced myofibre CSA^21^, ^38^, ^39^ associated with degeneration and structural alterations of the NMJ,^40^, ^41^, ^42^ a decline in functional innervation (partial denervation)^41^, ^43^, ^44^ and loss of motor units.^3^, ^44^, ^45^ Although it is not justified to extrapolate results from transgenic animal models to human muscles (i.e. to assume that each fibre type exhibits similar age‐related phenotypic changes with ageing in different species), the similar characteristics observed during neuromuscular ageing in both rodents and humans suggest that murine models can provide useful experimental models to explore the processes and mechanisms that contribute to skeletal muscle atrophy and weakness.

An understanding of the underlying causes of muscle atrophy and functional deficits inherent to ageing is critical for the development of strategies and targeted interventions to preserve the age‐related decline in neuromuscular integrity and function. This review summarizes the transgenic approaches that have been undertaken in rodent models to assess whether redox homeostasis is implicated in the processes of sarcopenia, unravel potential mechanisms involved in skeletal muscle ageing and identify areas where further research is warranted. We begin with a brief overview of the chemistry of RONS, sites of production and the antioxidant defence systems expressed in skeletal muscle, followed by a discussion of the redox sensitive pathways and cellular functions controlled by redox homoeostasis. This will be followed by a detailed discussion of the implication of redox homeostasis in neuromuscular ageing and the genetic modifications that have been undertaken to examine the potential link between redox control and age‐related deficits in skeletal muscle mass and function. Although we will discuss a broad range of topics related to the muscle redox environment, it is impossible to address all aspects of this expansive field of study in the present review. For topics not covered in detail in this article, we provide references of review articles where necessary.

## Skeletal muscle produces reactive oxygen and nitrogen species

**Figure 2:**
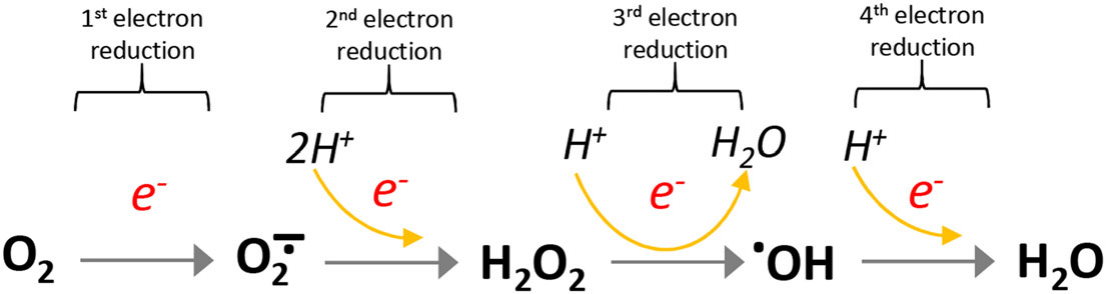
Reactive oxygen derivatives produced by the sequential reduction of O_2_ to H_2_O. Superoxide (O_2_∸), hydrogen peroxide (H_2_O_2_) and hydroxyl radical (^●^OH).

Molecular oxygen (O_2_) is one of the most abundant elements in the atmosphere (nearly 21% by volume), and its ability to accept electrons makes it vital for a variety of physiological processes. Aerobic organisms including humans have adapted well to the atmosphere, using atmospheric O_2_ by respiration and to obtain energy efficiency.^46^ Although O_2_ plays a key role in aerobic cellular metabolism, studies in the 1950s showed that O_2_ could cause cellular damage^47^ by the generation of reactive species (Figure 2), derivatives of O_2_.^48^ The ‘free radical theory of O_2_ toxicity’ sparked the interest of many research laboratories in the field of redox homeostasis in biological systems and the first studies to report that skeletal muscle produces reactive species appeared in the late 1970s^49^ and early 1980s.^50^ Over the past three decades, the field of redox biology has expanded rapidly, and with the development of high throughput ‘Omics’ technologies and sensitive analytical approaches, it is now widely accepted that resting and contracting skeletal muscle produces RONS both *in vivo* and *in vitro*.

Reactive oxygen and nitrogen species generation by myofibres has been detected and quantified by a variety of methods including high‐performance liquid chromatography techniques,^30^, ^51^ electron‐spin resonance spectroscopy (also known as electron paramagnetic resonance),^52^, ^53^ fluorescence‐based microscopic assays,^54^, ^55^ spectrophotometry,^56^, ^57^ chemiluminescence^58^, ^59^ and transfection methods including *in vivo* 60, 61 and *in vitro.* 62 It is widely accepted that superoxide and nitric oxide (NO) are the primary radical species generated by skeletal muscle.^46^, ^63^ Table 1 depicts the molecular formulas, half‐lives and intracellular concentrations of the major RONS produced by skeletal muscle. A discussion of the primary and ‘secondary’ RONS follows.

**Table 1:** Major RONS detected in skeletal muscle, estimates of half‐lives and cellular concentrations

Species	Formula	Biological half‐life(s)	Estimate cell conc. (M)	References
Superoxide	O_2_∸	10^−6^	1–10^−12^	64, 65, 66
Hydrogen peroxide	H_2_O_2_	10^−5^	1–10^−8^	64, 67, 68
Hydroxyl radical	^●^OH	10^−9^	ND	64, 69
Nitric oxide	NO	1–10^−1a^	1–10^−9^	66, 70, 71
Peroxynitrite	ONOO^−^	10^−2^	2^−9^	72, 73, 74

Not determined (ND).

NO half‐life depends on its concentration.

## Chemistry of reactive oxygen and nitrogen species produced by skeletal muscle

### Superoxide

**Figure 3:**
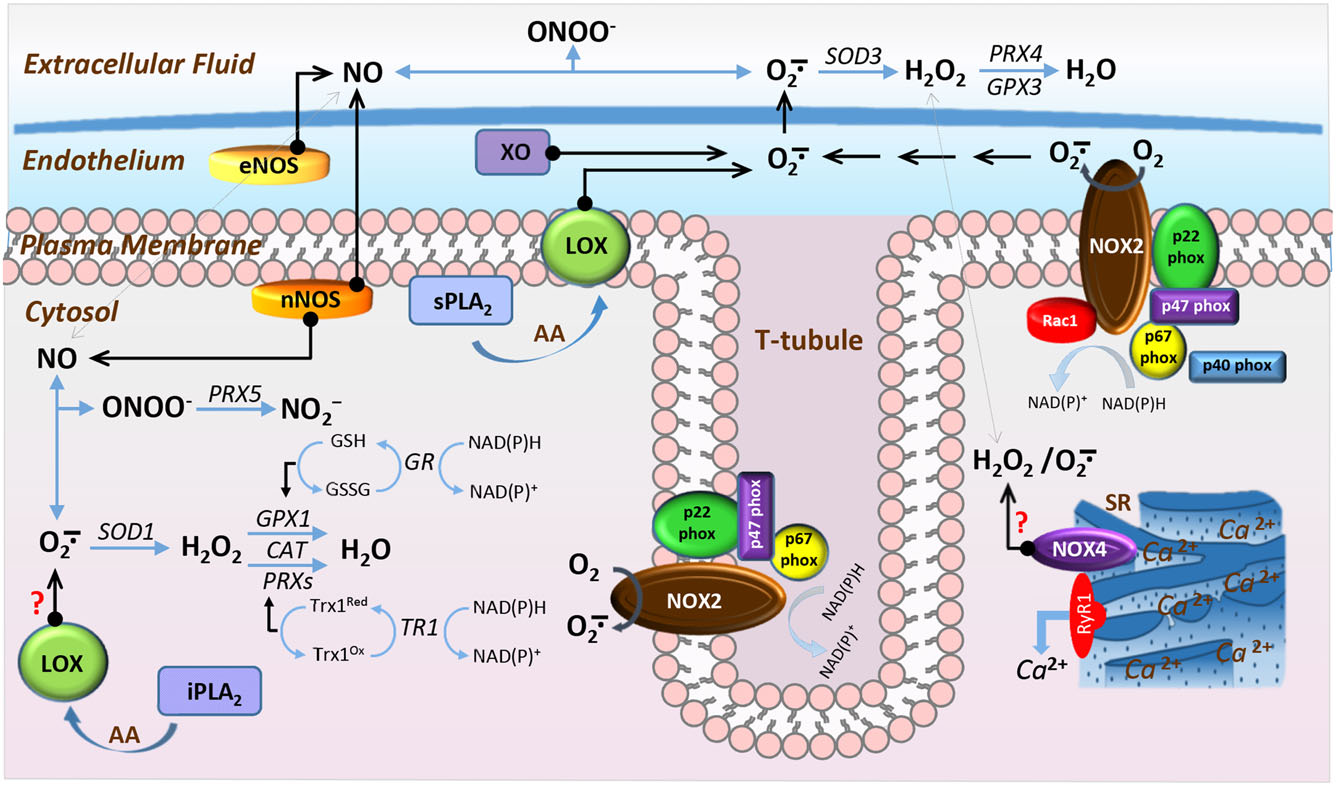
Schematic representation of the non‐mitochondrial sites for nitric oxide and superoxide production in skeletal muscle. Superoxide (O_2_∸) is produced by multicomponent nicotinamide adenine dinucleotide phosphate (NADPH) oxidase 2 (NOX2), xanthine oxidase and the lipoxygenases (LOX), which activity is regulated by the phospholipase A_2_ enzymes (PLA_2_). Arachidonic acid (AA) release by the membrane bound calcium‐dependent PLA_2_ (sPLA_2_) facilitates extracellular O_2_∸ release by the membrane bound LOX. It is uncertain whether the cytosolic LOX enzymes contribute to intracellular O_2_∸ changes, which substrate availability might be regulated by the cytosolic calcium‐independent PLA_2_ (iPLA_2_). NAD(P)H oxidase 4 (NOX4) also contributes to ROS changes, although the primary ROS product, O_2_∸, or hydrogen peroxide (H_2_O_2_) of NOX4 is uncertain. Cytosolic and extracellular O_2_∸ is dismuted into H_2_O_2_ by superoxide dismutase (SOD), SOD1 and SOD3, respectively, or reacts rapidly with membrane permeant nitric oxide (NO) produced by the endothelial and neuronal nitric oxide synthase (eNOS and nNOS) to form peroxynitrite (ONOO^−^). H_2_O_2_ formed within the extracellular space is reduced into H_2_O by the action of glutathione peroxidase 3 (GPX3) or peroxiredoxin IV (PRX4), while cytosolic H_2_O_2_ is reduced into H_2_O by glutathione peroxidase 1 (GPX1), catalase (CAT) or peroxiredoxins (PRXs). Reduced glutathione (GSH) provides the electrons to GPX to catalyse the reduction of H_2_O_2_; GSH is oxidized to glutathione disulfide (GSSG). Reduction of GSSG is catalysed by glutathione reductase (GR), where NADPH is used as the reducing agent. Cytosolic PRXs utilize thioredoxin 1 (Trx1^Red^) for their reducing action. Oxidized form of Trx1 (Trx1^Ox^) is reduced by thioredoxin reductase 1 (TR1) by utilizing electrons from NAD(P)H. ONOO^−^ can be reduced predominantly into nitrite (NO2^−^) by peroxiredoxin V (PRX5). Sarcoplasmic reticulum (SR).

**Figure 4:**
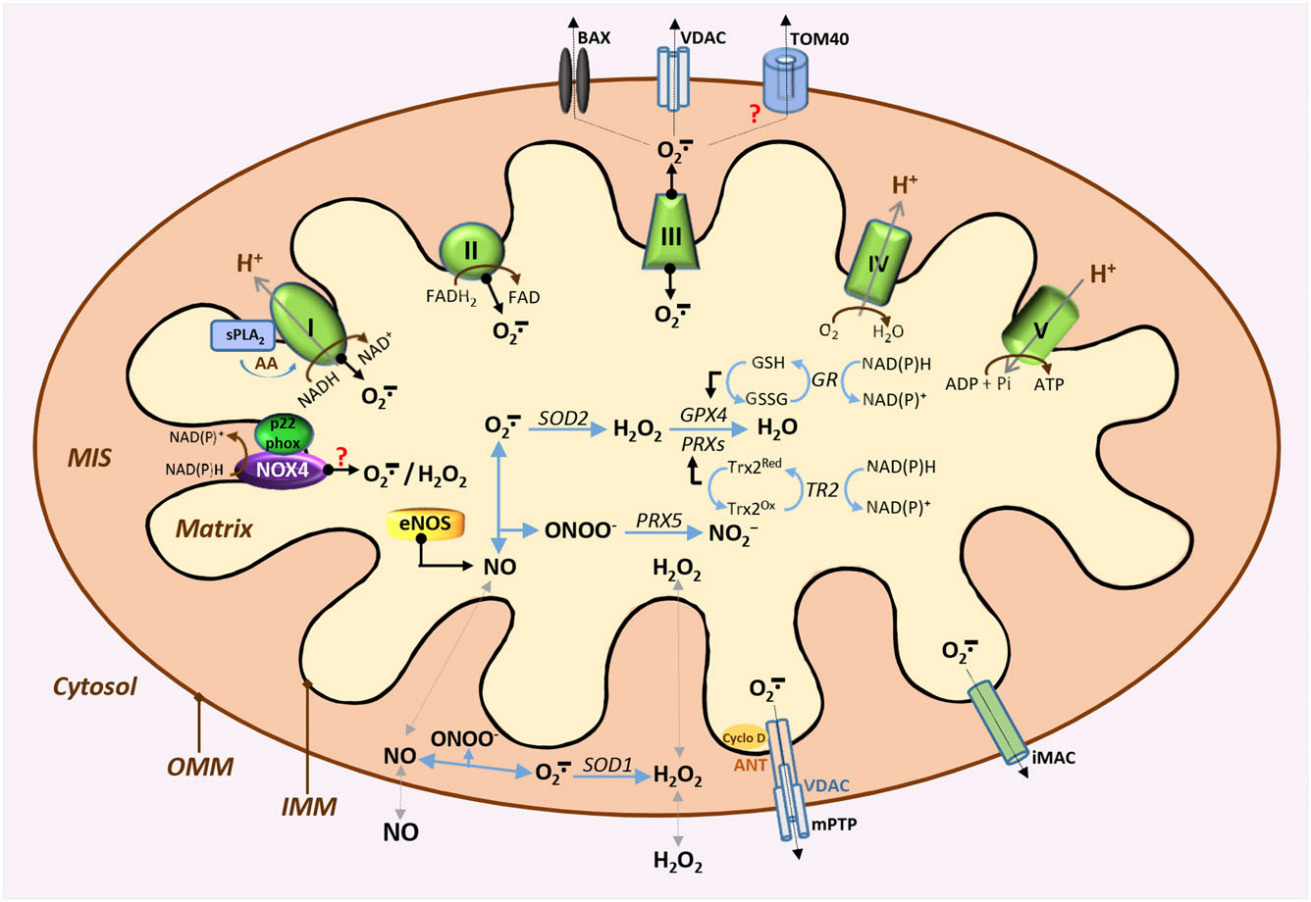
Schematic representation of the mitochondrial sites for nitric oxide and superoxide production and the channels that mediate the release of superoxide to the cytosolic compartment in skeletal muscle. Superoxide (O_2_∸) is produced by complex I, complex II and complex III of the mitochondrial electron transport chain of the inner mitochondrial membrane (IMM) and released into the matrix and the mitochondrial intermembrane space (MIS). Nicotinamide adenine dinucleotide phosphate (NADPH) oxidase 4 (NOX4) also contributes to ROS changes, although the primary ROS product, O_2_∸, or hydrogen peroxide (H_2_O_2_) of NOX4 is uncertain. Arachidonic acid (AA) release by the calcium‐dependent phospholipase A_2_ enzymes (sPLA_2_) interacts with complex I and enhances superoxide generation by this complex. O_2_∸ released into the matrix, and MIS is dismuted into H_2_O_2_ by superoxide dismutase (SOD), SOD2 and SOD1, respectively, or reacts rapidly with nitric oxide (NO) produced by the endothelial nitric oxide synthase (eNOS) to form peroxynitrite (ONOO^−^). H_2_O_2_ is reduced into H_2_O by the action of glutathione peroxidase 4 (GPX4) or peroxiredoxins (PRXs). Reduced glutathione (GSH) provides the electrons to GPX4 to catalyse the reduction of H_2_O_2_; GSH is oxidized to glutathione disulfide (GSSG). Reduction of GSSG is catalysed by glutathione reductase (GR), where NADPH is used as the reducing agent. Mitochondrial PRXs utilize thioredoxin 2 (Trx2^Red^) for their reducing action. Oxidized form of Trx2 (Trx2^Ox^) is reduced by thioredoxin reductase 2 (TR2) by utilizing electrons from NADPH. ONOO^−^ can be reduced predominantly into nitrite (NO2^−^) by peroxiredoxin V (PRX5). O_2_∸ is essentially membrane impermeant, while H_2_O_2_ is readily diffusible. Matrix O_2_∸ can diffuse to the cytosol through the inner membrane anion channel (iMAC) that spans the IMM and the outer mitochondrial membrane (OMM) or via the mitochondrial permeability transition pore (mPTP) composed of the voltage‐dependent anion channels (VDAC) on the OMM, the adenine‐nucleotide translocator (ANT) located on the IMM and cyclophilin D (Cyclo D) located in the matrix. Channels of the OMM including VDAC, BAX and possibly the translocase of outer membrane 40 (TOM40) can also mediate the release of O_2_∸ from the MIS to the cytosol.

Superoxide is one of the main radical species produced by skeletal muscle and is derived either from incomplete reduction of O_2_ in electron transport systems or as a specific product of enzymatic systems.^75^ Resting and contracting skeletal muscle produces superoxide via different pathways, and schematic Figures 3 and 4 depict the various sites and mechanisms that have been proposed for RONS generation in skeletal muscle. Briefly, superoxide is generated by the mitochondrial electron transport chain including complex I, complex III^76^, ^77^ and, recently, complex II^78^, ^79^, ^80^; the nicotinamide adenine dinucleotide phosphate (NADPH) oxidase enzymes including NOX2, NOX4, DUOX1 and DUOX2^55^, ^56^, ^59^, ^81^; xanthine oxidase^82^, ^83^; and the lipoxygenases (LOXs),^84^ which are linked to arachidonic acid released by the phospholipase A_2_ enzymes^85^, ^86^ (for a detailed review, see Ref. [46].

Superoxide anion carries a negative charge and cannot diffuse through membranes. However, it can cross membranes through anion channels including the inner membrane anion channel and the voltage‐dependent anion channels^55^, ^87^, ^88^ (Figure 4). Nonetheless, it has been argued that superoxide can be protonated at physiological pH to produce the hydroperoxyl radical, enabling the transfer of superoxide across biomembranes.^89^ Although superoxide anion has a relatively long half‐life, it has a limited oxidizing ability as it does not react directly with polypeptides, sugars or nucleic acids but can interact with other molecules to generate secondary RONS either directly or through enzyme or metal‐catalysed processes.^65^ In aqueous solutions, dismutation of superoxide into hydrogen peroxide (H_2_O_2_) can occur spontaneously or catalysed by superoxide dismutases (SODs)^90^ with a rate constant (*k* = 2 × 10^9^ M^−1^ s^−1^),^70^ a reaction considered to be very slow as superoxide radicals electrostatically repel each other.^91^

### Hydrogen peroxide

Hydrogen peroxide (H_2_O_2_) is a relatively stable molecule with a long half‐life and can diffuse across biomembranes.^92^ H_2_O_2_ has been suggested to be a redox signalling molecule^93^ that can interact with redox‐sensitive components or pathways, activating various transcription factors in skeletal muscle.^94^ In addition to SOD‐dependent production of H_2_O_2_, a number of enzyme systems also generate H_2_O_2_ including NOX4,^95^, ^96^ urate and amino acid oxidases.^97^ Moreover, recent evidence supports endoplasmic reticulum (ER) H_2_O_2_ generation *in vivo* 98 via thiol‐disulfide exchange mechanisms.^99^ Elevated concentrations of H_2_O_2_ have shown to alter the catalytic activity of enzymes by oxidizing thiol groups of essential amino acids^100^; cytotoxicity of H_2_O_2_ in skeletal muscle occurs through the generation of hydroxyl radicals via metal‐catalysed reactions.^101^

### Hydroxyl radical

Hydroxyl radicals have a strong oxidizing potential with a half‐life in aqueous solution of less than 1 ns^69^ and can react rapidly with almost any biomolecule close to their site of production. Hydroxyl radicals occur in skeletal muscle fibres from the reductive decomposition of H_2_O_2_ with reduced transition metal ions, iron (Fe) or copper (Cu), through a reaction called the Fenton reaction.^102^ There is some controversy over the Fenton reactions particularly *in vivo* due to the concentration of reactive transition metal ions being very low^103^ and its small rate constant (*k* = 10^9^–10^10^ M^−1^ s^−1^).^104^ There is, however, evidence that disrupted redox homeostasis can lead to oxidation of Fe cluster‐containing enzymes, thus releasing ‘free Fe’ enabling hydroxyl radical formation.^105^ Hydroxyl radical generation is also facilitated by the Haber–Weiss reaction that makes use of Fenton chemistry, in which Fe or Cu is maintained in a reduced form by superoxide, thus capable of catalysing the generation of hydroxyl radicals from H_2_O_2_.^106^ Hydroxyl radicals are membrane impermeant, and evidence has shown enhanced generation of hydroxyl radicals *in vivo* during muscle contractile activity.^107^ Enhanced generation of hydroxyl radical formation can affect calcium (Ca^2+^) sensitivity and maximum force of skeletal myofibres^102^; further reports have identified increased generation of hydroxyl radicals in neuromuscular disorders including glucocorticoid‐induced myopathy^101^ and immobilization‐induced skeletal muscle atrophy.^108^

### Nitric oxide

Nitric oxide, also known as nitrogen monoxide, is a primary radical and arises through the conversion of l‐arginine to citrulline by the NO synthases (NOS), utilizing NADPH as a cofactor.^109^ NO is a weak reducing agent, with a relatively long half‐life^70^ and reacts with O_2_ to form nitric dioxide and superoxide to produce peroxynitrite.^110^ There are three different isoforms of NOS expressed in skeletal muscle; the neuronal NOS (nNOS or type I), the inducible NOS (iNOS or type II) and the endothelial NOS isoenzyme (eNOS or type III).^46^, ^111^ nNOS, originally discovered in neuronal tissue, is expressed along the sarcolemma of skeletal muscle fibres and interacts with the dystrophin–glycoprotein complex via a linkage to α1‐syntrophin.^112^ Type III NOS isoenzyme, originally described in the endothelium through association with caveolin‐1, is localized in the muscle mitochondria and is activated through association with heat shock protein 90.^113^ Inducible NOS isoenzyme is involved in the immune response and is primarily expressed in skeletal muscle in response to inflammatory conditions or a septic challenge.^114^, ^115^ NO has shown to regulate cytoskeletal proteins,^116^ and nNOS isoform, strongly expressed in glycolytic muscle fibres,^117^ has been reported as the prime source of NO release from skeletal muscle.^118^ The importance of NO signalling in muscle physiology is highlighted in mdx mice^119^ and humans with Duchenne muscular dystrophy.^112^, ^120^ NO responses are largely mediated via cysteine (Cys) *S*‐nitrosylation or by coordinated interactions with heme or non‐heme Fe and Cu.^121^

### Peroxynitrite

Peroxynitrite, a powerful oxidant with a relatively long half‐life, is produced through the reaction of NO with superoxide.^122^ Evidence in skeletal muscle has shown *in vivo* intracellular generation of peroxynitrite in myofibres of transgenic murine models in which the levels of superoxide and/or NO were up‐regulated.^30^ The chemical reaction of superoxide with NO to generate peroxynitrite has a reaction rate (*k* = 7 × 10^9^ M^−1^ s^−1^),^70^ which is approximately three‐fold higher than the SOD catalysed conversion of superoxide to H_2_O_2_ (*k* = 2 × 10^9^ M^−1^ s^−1^) as previously discussed. Peroxynitrite can react with thiol compounds to form disulfides^123^ and, along with its protonated form, peroxynitrous acid, can deplete thiol groups and induce protein, phospholipid oxidation and DNA damage.^92^, ^122^ Peroxynitrite leads to nitration of tyrosine residues,^124^ and *S*‐nitrosylation of Cys residues,^125^ the list of proteins being nitrated and nitrosylated in skeletal muscle, is continuously growing. Under circumstances where peroxynitrite is generated at high concentrations, it can not only cause oxidative damage to cellular compartments of myofibres^18^, ^30^ but also alter the structure and function of proteins resulting in altered catalytic activity of enzymes, altered cytoskeletal organization and impaired cell signal transduction.^122^

### Redox regulation in skeletal muscle

Over the last three decades, it has become clear that RONS can act as mediators of contraction‐induced damage to skeletal muscle.^68^ Muscle cells contain a network of antioxidant defence mechanisms to control the cellular production of RONS and maintain the redox environment. The antioxidant network includes enzymatic and non‐enzymatic systems, and the potential role of redox homeostasis as the underlying factor implicated in neuromuscular ageing has been the subject of intensive research in a variety of model organisms. The important technological advances that have occurred in the last few decades have allowed research groups to utilize genetic engineering techniques to alter specific genes or crucial redox components of the antioxidant network and assess whether age‐related deficits in neuromuscular integrity and function are mediated by defective redox signalling. Figures 3 and 4 depict the subcellular RONS protective systems expressed in skeletal muscle. Description of the antioxidant mechanisms follows.

## Regulatory reactive oxygen and nitrogen species enzymes expressed in skeletal muscle

### Superoxide dismutase

Superoxide dismutase found in all O_2_‐utilizing organisms catalyses the dismutation of superoxide to H_2_O_2_ and O_2_.^126^ Three isoforms of SOD that exist are mammalian skeletal muscle depending on cellular location and the redox active transition metal bound to its active site to accomplish the catalytic breakdown of superoxide^30^, ^46^; copper–zinc SOD (SOD1 or CuZnSOD), localized within the mitochondrial intermembrane space (MIS) and cytosol, requires Cu–Zn as a cofactor; and manganese (Mn) SOD (SOD2 or MnSOD) requires Mn as a cofactor and is expressed in the mitochondrial matrix.^92^ Extracellular SOD isoenzyme incorporates Cu–Zn as a cofactor and is present in extracellular fluids and interstitial spaces of tissues.^127^ Evidence has shown that exercise can induce an increase in both SOD1 and SOD2 activities in skeletal myofibres.^128^ SOD1 protein has a half‐life of 6–10 min, whereas SOD2, 5–6 h.^129^ Fifteen to thirty‐five per cent of the total SOD activity resides within the mitochondria of skeletal muscle, with the SOD2 isoenzyme accounting for 15–20%,^130^ and the remaining 65–85% remains within the cytosolic compartment of muscle cells.^131^ SOD1 and SOD2 protein expression and activity are higher in oxidative muscle fibres compared with those of fast glycolytic fibres.^103^

### Glutathione peroxidase

Glutathione peroxidase (GPX) catalyses the reduction of H_2_O_2_ or organic hydroperoxide to H_2_O and alcohol, respectively, using reduced glutathione (GSH) or in some cases glutaredoxin (GRX) or thioredoxin (TRX) as an electron donor.^92^ Five GPX isoforms are reported in mammals, which differ in cellular localization and substrate specificity with GPX1 localized predominantly in the cytosol and a small proportion in the mitochondrial matrix. Seleno‐protein GPX4, a membrane‐associated enzyme, is partly localized to the MIS, while GPX3 is present in the extracellular space.^132^ Although the GPX antioxidant system has not been as extensively described as other antioxidant systems (e.g. SOD redox network), GPX gene expression is controlled by a range of mechanisms including toxins, O_2_ tension, metabolic rate and growth and development.^65^ The relative amounts of GPX expressed in skeletal muscle are higher in oxidative fibres compared with fibres with low oxidative capacity,^75^ and exercise has shown to up‐regulate the protein expression and activity of both cytosolic and mitochondrial GPX in skeletal muscle fibres.^131^

### Catalase

Catalase (CAT) is distributed within the cytosolic compartment of myofibres and catalyses the breakdown of H_2_O_2_ into H_2_O and O_2_.^133^ Fe is a required co‐factor, bound at the enzyme's active site for its catalytic function.^134^ Although GPX is also an H_2_O_2_ regulator in skeletal muscle and they share common substrates, CAT has a lower affinity for H_2_O_2_ at low concentrations (*K*_m_ = 1 mM) compared with GPX (*K*_m_ = 1 μM).^135^ In situations where H_2_O_2_ is significantly elevated, CAT becomes an important H_2_O_2_ reducing system, and its enzymatic activity prevails because there is no apparent *V*_max_ and cannot be saturated by H_2_O_2_ at any concentration.^136^ CAT enzymatic activity is higher in oxidative myofibres compared with fast glycolytic fibres^137^ and does not require reducing equivalents to function as a H_2_O_2_ reducer; thus, CAT is considered an energy‐efficient enzyme.^138^

### Peroxiredoxins

The family of peroxiredoxins (PRXs) initially known as thiol‐specific antioxidants^139^ are Cys‐dependent TRX peroxidases that are capable of reducing both H_2_O_2_ and organic hydroperoxide.^140^ In two Cys peroxiredoxins (2‐Cys PRXs), on reaction with H_2_O_2_, the redox‐sensitive Cys residue of each subunit of the PRX homodimer is oxidized to Cys‐SOH, which then reacts with a neighbouring Cys‐SH to form an intermolecular disulfide.^141^ It is noteworthy that PRXVI possesses only one Cys residue in the active site, while other PRXs contain 2‐Cys PRXs.^142^ The intramolecular disulfide of 2‐Cys PRXs is reduced specifically by electrons provided by TRXs, which are then regenerated by TRX reductase at the expense of NADPH,^143^ thus restoring the catalytic activity.

Six isoforms of PRX are expressed in skeletal muscle; PRX I, II and VI are localized in the cytosolic compartment, PRXIII exclusively in skeletal muscle mitochondria, PRXIV in the extracellular space and ER and atypical 2‐Cys PRXV in the cytosol, mitochondria, nuclei and peroxisomes.^46^, ^142^ All of the six mammalian PRX proteins act to degrade H_2_O_2_. PRXV has also reported to have peroxynitrite reductase activity,^144^ and PRXVI has shown to facilitate NOX1^145^ and NOX2^146^ optimal activities. PRX isoforms are highly abundant in skeletal muscle and have a high catalytic efficiency as peroxidases.^147^ However, a number of these isoforms can be inactivated, mediated by the oxidation of the catalytic site Cys to Cys‐sulfinic acid (SO_2_H) by high levels of H_2_O_2_. PRXs that have formed SO_2_H acids can be reduced by sulfiredoxin, and it has been proposed that this initial inactivation by its substrate plays a cellular signalling role by a ‘floodgate mechanism’.^148^ It is particularly interesting that there are a number of protein families and isoforms within those families that have either peroxidase activity (GPXs, PRXs and CAT) or regulatory proteins that are sensitive to oxidation (Keap1, aconitase and PTP1B). It is important to recognize that the rate constants within these protein families and isoforms can vary by up to 10^6^ M^−1^ s^−1^,^149^ suggesting that the concentration of H_2_O_2_ and the enzymes that regulate it play a significant role in redox signalling. PRX proteins have been shown to play a role in transmitting redox signals into a dynamic biological response and to have subtle changes in both abundance and oxidative state with age.^52^, ^150^, ^151^ PRXII has recently been found to form an interdisulfide with STAT3 in response to cytokines, suggesting that it plays an important regulatory role.^152^

### Thioredoxins

Thioredoxins are ubiquitous antioxidant enzymes that contain a canonical dithiol‐disulfide active site (Cys‐Gly‐Pro‐Cys),^153^ originally discovered in 1964 in Escherichia coli as an electron donor for ribonucleotide reductase.^154^ It has become clear that TRXs play multivalent cellular roles by serving as electron donors for enzymes such as ribonucleotide reductases, PRXs and methionine sulfoxide reductases (MSRAs),^155^ protecting proteins from oxidative aggregation and inactivation.^156^ Skeletal muscle expresses TRX1 (expressed in cytosol and nucleus) and TRX2 located within the mitochondrial compartment.^157^ The Cys residues of the Cys‐Gly‐Pro‐Cys motif are the key players used by TRXs to reduce disulfide bonds in oxidized substrate proteins and upon completion of a catalytic cycle; these two Cys residues are oxidized and form a disulfide.^156^ Oxidized Cys residues are converted back to the reduced state by TRs with TR1 isoform present in the cytosol and nuclei and TR2 in the mitochondria, at the expense of NADPH. TRXs have also been implicated in various cellular processes including protein structure/folding energy utilization, transcription factor regulation and immune/inflammatory response.^156^

### Glutaredoxins

Glutaredoxins are a family of thiol‐disulfide oxidoreductases that utilize the reducing power of GSH to catalyse disulfide reductions in the presence of NADPH and glutathione reductase (GR).^158^ Depending on the number of active site Cys residues, GRXs are divided into dithiol (Cys‐X‐X‐Cys) and monothiol (Cys‐X‐X‐Ser) GRXs.^159^ Dithiol GPXs conduct similar functions to the TRX system; they can participate in the regulation of H_2_O_2_ via PRX pathways,^160^ transcription regulation via modulating the activity of nuclear factor κB (NFκB),^161^ proliferation and differentiation^162^ and apoptosis.^163^ Monothiol GRXs function primarily both in the biosynthesis of FeS proteins and Fe homeostasis.^164^ GRX1 is mainly localized in the cytosol but can be found in the MIS; it can be translocated into the nucleus and exported from the cell.^159^ GRX2 is expressed in the mitochondria,^165^ GRX3 in the cytosolic and nuclear compartment, and monothiol GRX5 has a mitochondrial translocation signal and shares the active site motif of GRX3.^166^ Evidence has also shown that the GRX system can also catalyse reversible protein glutathionylation,^167^ which is an important redox regulatory mechanism, and control the redox state of thiol groups^168^ in situations where the redox environment is being compromised.

Additional enzymes expressed in skeletal muscle including isocitrate dehydrogenase and glucose‐6‐phosphate dehydrogenase are also involved in the antioxidant defence system by providing reducing power in the form of NADPH to the antioxidant enzymes.^169^ Although these enzymes do not directly scavenge RONS, their contribution to maintain the redox environment in myofibres is significant.

## Non‐enzymatic key antioxidants that contribute to the maintenance of muscle cellular redox state

A variety of non‐enzymatic antioxidants, endogenous and exogenous (through diet), are found in skeletal muscle and have shown to contribute to the maintenance of muscle redox status. These include not only GSH, bilirubin, uric acid and coenzyme Q_10_ that endogenously produced antioxidants but also dietary antioxidants including carotenoids, vitamin C and vitamin E. A detailed description of the non‐enzymatic defence mechanisms in skeletal muscle goes beyond the scope of this review; for a detailed description, see Refs [170, 171]. However, we provide a short overview of the main endogenously produced antioxidant, GSH, which plays an important role in maintaining the redox environment in skeletal muscle cells by directly reacting with RONS through a hydrogen atom donation or indirectly during GSH‐dependent peroxidase‐catalysed reactions,^172^ as previously discussed.

### Glutathione

The tripeptide GSH (l‐*γ*‐glutamyl‐l‐cysteinyl‐glycine) is synthesized in a two‐step process catalysed by glutamate‐Cys ligase (l‐glutamate:l‐Cys *γ*‐ligase) and glutathione synthetase (γ‐l‐glutamyl‐l‐Cys:glycine ligase).^65^ GSH is consumed in various ways, such as by oxidation, conjugation and hydrolysis. GSH can be directly oxidized by RONS, act as a substrate for GSH‐dependent enzymatic reactions and conjugate with endogenous and exogenous electrophiles.^172^ GSH is distributed to intracellular organelles including the ER, nucleus and mitochondria.^173^ GSH can react directly with a variety of radicals by donating a hydrogen atom and has shown to reduce vitamin E and C radicals derived in chain‐breaking reactions with lipid peroxyl or alkoxyl radicals.^171^

Mitochondrial organelles lack CAT; the reduction of H_2_O_2_ is accomplished mainly by GSH, with the participation of either GPX or PRX and by the later conversion of glutathione disulfide (GSSG) back into GSH by GR. Moreover, the GSH system is also associated with the GRX system and the removal of xenobiotics by glutathione *S*‐transferases. Under impaired redox homeostasis, a significant number of proteins can be altered in their function by formation of mixed disulfides and the GSH‐dependent disulfide oxidoreductase GRX system catalyses dithiol reactions, reducing GSH‐protein mixed disulfides in a coupled system with GR.^173^ Oxidative muscle fibres contain a higher GSH content compared with fast glycolytic fibres,^130^ although the ratio GSH/GSSG appears to be consistent across various fibre types.^174^ Myofibre GSH levels increase in response to exercise,^175^, ^176^ and high intracellular levels of GSSG have shown to inactivate enzymes and induce glutathionylation in skeletal muscle.^177^

### Skeletal muscle cysteine redox modifications and oxidative damage are regulated by reactive oxygen and nitrogen species

The first human study to report that exercise enhanced oxidative damage appeared in the late 1970s.^49^ This study instigated intensive research in the field or redox biology, and the first article to demonstrate that skeletal muscle augmented reactive species in response to contractile activity appeared in 1982.^50^ These studies cited the mitochondrial organelle as the major source of reactive species in muscle cells,^50^, ^178^ but over the past 35 years, the development of analytical approaches has been instrumental in the discovery of additional redox sites in various subcellular compartments of skeletal myofibres.^46^, ^55^ RONS produced by skeletal muscle were initially considered as ‘toxic’ by‐products of metabolic processes inducing cellular damage and since these initial reports, a substantial amount of evidence suggests that redox homeostasis plays an underlying role in various human myopathies, neurodegenerative and metabolic diseases including muscular dystrophies, amyotrophic lateral sclerosis, Alzheimer's disease, Parkinson's disease and diabetes; for detailed reviews, see Refs [112, 179, 180, 181].

The magnitude and species of RONS generated by skeletal muscle have downstream effects on specific protein targets and cellular redox signalling. Recent application of novel redox proteomic approaches has identified and quantified reversible and irreversible modifications of susceptible Cys residues of redox‐sensitive proteins expressed in skeletal muscle.^81^, ^151^, ^182^ An extended coverage of these goes beyond the scope of this review; for a detailed description, see Refs [183, 184, 185]. Briefly, the type of redox modification on Cys residues depends on the concentration and species of RONS as well as the amino acids surrounding the Cys residue. Reversible modifications of Cys residues include glutathionylation, nitrosylation, sulfenylation (─SOH) and inter/intradisulfide bond formation.^183^ The largely irreversible modifications include SO_2_H and sulfonic (SO_3_H) acids.^184^

Contraction‐induced RONS by skeletal muscle has shown to contribute to muscle fatigue^186^; induce oxidative damage including lipid peroxidation, protein oxidation and DNA damage^66^; and alter the function of redox‐sensitive proteins within myofibres.^151^ The sensitivity of a particular target is defined by the intrinsic sensitivity of the molecule to oxidation–reduction and the local redox state,^103^ and evidence has shown that RONS produced by skeletal muscle can alter myofilament structure and function.^187^ Several myofilament proteins including actin, α‐actinin,^151^, ^187^ troponin C^188^ and myosin heavy chains^189^, ^190^, ^191^ are susceptible to RONS‐induced oxidative modifications, thus affecting Ca^2+^ dynamics and Ca^2+^ sensitivity^192^ and, inevitably, cross‐bridge kinetics,^188^ which may result in contractile dysfunction.

Abundant evidence further indicates that altered muscle redox environment due to elevated RONS production by skeletal muscle fibres is implicated in muscle atrophy induced by muscle disuse^193^ and disease.^194^ The causative links between redox homeostasis and muscle atrophy induced by skeletal muscle inactivity were recently reviewed^195^, ^196^, ^197^ and include reduced anabolic signalling and protein synthesis via inhibition of Akt/mTORC1 signalling, elevated proteolytic pathways including enhanced autophagy, activation of calpains and caspase‐3 and increased protein breakdown via the proteasome system.

### Skeletal muscle reactive oxygen and nitrogen species are required for multiple intracellular signalling pathways and cellular functions

Although it is widely accepted that RONS produced by skeletal muscle can induce oxidative damage and alter muscle physiology, in situations when the antioxidant system is compromised or when RONS are excessively augmented, abundant evidence indicates that the redox environment plays an important role in modulating multiple signalling pathways and muscle cellular functions.^112^, ^198^, ^199^, ^200^, ^201^, ^202^, ^203^ The advantageous biological effects of RONS in muscle physiology contradict early reports that RONS are by‐products of metabolism, inevitably damaging to muscle cells. A detailed discussion of RONS‐dependent signal transduction pathways is beyond the scope of this review, but examples of key biochemical pathways and cellular processes that require a particular ‘optimal redox state’ in skeletal muscle are provided in Table 2. The findings depicted in Table 2 highlight that physiological levels of RONS are essential and play crucial roles in regulating skeletal muscle metabolism and physiology. Deciphering the mechanisms that underlie the divergence between adaptive and maladaptive responses to RONS in skeletal muscle remains an active area of research.^226^

**Table 2:** Redox sensitive pathways/processes in skeletal muscle metabolism and physiology

Redox‐sensitive cellular functions and biochemical pathways	References
•Regulation of Ca^2+^ release from the sarcoplasmic reticulum (SR) via a ryanodine receptor Ca^2+^ release redox mechanism.	56, 81
•Ca^2+^ sensitivity of myofilaments via oxidative modifications of the amino acids in the Ca^2+^ binding sites of cytoskeletal proteins that alter optimum troponin Ca^2+^ binding and actin myosin interactions.	204, 205
•Regulation of muscle force production.	202, 206, 207
•Activation of redox sensitive transcription factors including NFκB, AP‐1 (activator protein 1), HSF‐1 (heat‐shock factor 1), Nrf2 (nuclear factor erythroid 2‐related factor) and gene expression.	208, 209, 210, 211
•Modulation of contractile activity‐dependent increase in RONS regulatory protein expression and HSP content.	212, 213, 214
•Activation of key signalling molecules such as PGC1*α* (peroxisome proliferator‐activated receptor α), AMPK (AMP‐activated protein kinase) and MAPK (mitogen‐activated protein kinase), which regulate cellular mechanisms for muscle adaptation (e.g. oxidative metabolism and mitochondrial biogenesis/function).	198, 211, 215, 216
•Induction of signalling cascades for autophagy or apoptosis under physiological conditions.	198
•Modulation of gene expression of mitochondrial transcription regulators, Sirtuin 1 and mitochondrial biogenesis.	217
•Regulation of ion channels, protein phosphatases and kinases that modulate the activity of various enzymes involved in oxidative phosphorylation, tricarboxylic acid cycle and glycolysis.	121, 218, 219
•Regulation of contraction‐stimulated glucose uptake in skeletal muscle via RONS signalling.	220, 221, 222, 223
•Modulation of protein synthesis via the IGF‐1 (insulin‐like growth factor 1) signalling pathway.	224, 225

### Age‐related deficits in skeletal muscle mass and function are associated with altered redox homeostasis

Identification of the mechanisms underlying the structural and functional changes that occur in skeletal muscle during ageing has stimulated the interest of many laboratories with a goal of identifying pharmaceutical targets to combat physical frailty and mobility impairment that affect up to half the population aged 80 or older.^32^ It has been 60 years since Denham Harman proposed the ‘free radical theory of ageing’.^227^ Although it is now recognized that this theory and its various derivatives do not exclusively explain the ageing process,^228^, ^229^ disrupted redox signalling has been suggested to be implicated in the processes of loss of neuromuscular integrity and function that occurs during ageing.^230^

Skeletal muscle decline with advancing age has been linked to an altered oxidative status of redox‐responsive proteins,^183^ a positive correlation between tissue concentration of oxidized macromolecules and lifespan including an increase in DNA damage,^14^, ^231^ accumulation of oxidized proteins^15^, ^16^ and increased levels of lipid peroxidation^232^, ^233^ in both humans and rodents. Recent quantitative proteomic approaches have further provided evidence that muscle ageing is associated with altered catalytic activity of regulatory enzymes and a reduction in detection of redox‐sensitive proteins involved in the generation of precursor metabolites and energy metabolism,^151^, ^183^ implying age‐related redox changes as an underlying cause of skeletal muscle ageing.

Based on findings in the early 1970s that mitochondria can generate reactive species,^234^ a variant of the free radical theory of ageing, the ‘mitochondrial free radical theory of ageing’ was proposed.^235^ Consistent with a role of mitochondria as a contributor to age‐related muscle redox changes, reports have shown that isolated^213^, ^236^ and intact mitochondria^21^ in skeletal muscle fibres exhibit an age‐dependent increase in H_2_O_2_ generation. Considerable evidence has shown that age‐related mitochondrial oxidative damage can alter mitochondrial integrity and function in ageing skeletal muscle. Several key features have been observed in ageing skeletal muscle, including a reduction in mitochondrial abundance^237^ and oxidative‐phosphorylation,^19^ accumulation of mutated mtDNA^238^ associated with impaired mitophagy,^21^, ^38^ increased mitochondrial permeability transition pore sensitivity^54^ and increased mitochondrial‐mediated apoptosis,^239^ which collectively may contribute to age‐related loss of neuromuscular integrity and function. Together, these findings may support the conclusion that age‐related muscle atrophy and functional deficits are associated with increased oxidative damage and defective redox signalling.

The relationship between redox homeostasis and neuromuscular ageing has been further examined in several mammalian models that have undergone genetic manipulations, to enable the study of aberrant redox homeostasis on the ageing process.

### Redox homeostasis and age‐related deficits in neuromuscular integrity and function, insights from transgenic animal models

In the last two decades, the field of redox biology has advanced significantly with the development of new analytical approaches and techniques in genetic manipulation. The impact of altered redox homeostasis in loss of neuromuscular integrity and function with ageing has been investigated in several murine models, which have undergone genetic modifications of redox signalling/homeostasis components.^19^, ^23^, ^25^, ^29^, ^30^, ^31^, ^32^, ^33^, ^34^, ^240^, ^241^, ^242^ Transgenic murine models have provided insight into the importance of RONS regulatory systems in lifespan and neuromuscular ageing, and it has been reported that SOD2^−/−^,^243^ GRX3^−/−^,^244^ GPX4^−/−^,^245^ TRX1^−/−^,^246^ TRX2^−/−^,^247^ TR1^−/−^ 248 and TR2^−/−^ 249 murine models are embryonically lethal. Although the embryonic lethal phenotypes observed in these specific knockout models do not facilitate our understanding on whether defects in redox signalling affect age‐dependent deficits in neuromuscular integrity and function, these findings, however, highlight the fundamental importance of the redox systems mentioned in the preceding text during embryonic development.

SOD1^−/−^,^27^ PRX1^−/−^,^250^ PRX2^−/−^,^251^ TRX2^+/−^ 252 and MSRA^−/−^ 253 rodent models show a reduction in lifespan; in contrast, more recent studies have reported no effect of MSRA^−/−^ on rodent lifespan.^254^ GPX1^−/−^,^28^, ^252^ SOD2^+/−^,^240^ extracellular SOD^−/−^ 255, 256 and MSRB^−/−^ 257 knockout murine models show no effect on lifespan. Similarly, transgenic animal models overexpressing RONS protective enzymes including SOD1^Tg^,^26^ SOD2^Tg^,^24^ MSRA^Tg^,^258^ mice overexpressing human CAT in nuclei (nCAT^Tg^)^259^ and peroxisomal targeted CAT (pCAT^Tg^) (the natural site of CAT)^260^ have failed to provide evidence of increased lifespan, indicating that RONS are not the fundamental determinants of lifespan. However, GPX4^+/−^,^261^ TRX1^Tg^ 262 and the mitochondrial CAT overexpressing (mCAT^Tg^) mouse model^263^ showed ~7%, ~14% and ~21% increases in lifespan, respectively, which may provide support for the theory of oxidative damage in ageing.

It is noteworthy that the majority of genetic interventions in mice has been undertaken in C57BL/6, the most widely used inbred strain. Recently, it was suggested that this particular strain might not be suitable to study the effect of redox homeostasis in ageing due to a missense mutation in the nicotinamide nucleotide transhydrogenase protein that links the NAD/NADH to NADP/NADPH pool, providing reducing equivalents for TRX reductase and GRX redox enzymes.^264^ Table 3 summarizes the genetically engineered rodent models that have been developed to assess the implication of redox homeostasis in age‐related deficits in neuromuscular integrity and function.

**Table 3:** List of studies that have manipulated RONS regulatory systems to investigate the effect of redox homeostasis in age‐related deficits in neuromuscular integrity and function

Model	Neuromuscular phenotype and function	References
**Mitochondrial redox systems**		
SOD2^+/−^	•No effect on age‐related neuromuscular ageing •increased RONS generation in skeletal muscle and elevated mitochondrial oxidative damage •defective signalling in the PI3‐Akt pathway •impaired phosphorylation of Akt at Ser473 and Thr308 and decreased differentiation potential •reduced treadmill endurance capacity.	24, 265, 266, 267, 268, 269
TnIFastCre SOD2^fl/fl^	•No effect on age‐related neuromuscular ageing •increased mitochondrial RONS and oxidative damage •complex II‐linked mitochondrial dysfunction •reduced contractile muscle function and aerobic exercise capacity.	25, 270
SOD2^Tg^	•No effect on age‐related muscle atrophy •preserved mitochondrial mass and function •preserved the differentiation potential • no changes in RONS production in resting skeletal muscle myotubes.	24, 268
mGPX4‐KO	•No effect on age‐related neuromuscular ageing.	271
PRX3^−/−^	•No effect on muscle atrophy or skeletal muscle isometric force •increased mitochondrial RONS and altered mitochondrial membrane potential and network •decreased mitochondrial DNA, ATP production, mitofusin 1 and 2 protein levels • increased muscle fatigue resistance.	272
mCAT^Tg^	•No effect on age‐related muscle atrophy or fibrosis •reduced mitochondrial oxidative damage and insulin resistance •preserved mitochondrial respiration and ATP synthesis •prevented age‐related reduction in AMP‐activated protein kinase •improved complex I respiratory dysfunction •improved voluntary exercise and increased skeletal muscle specific force and tetanic Ca^2+^ transients •decreased intracellular Ca^2+^ leak and increased sarcoplasmic reticulum Ca^2+^ load.	19, 32, 242
**Other redox systems**		
nNOS^Tg^	•No effect on age‐related muscle atrophy or muscle weakness •prevented muscle membrane injury and reduced muscle inflammation following a hindlimb muscle unloading and reloading protocol •increased protein nitration.	30, 273
GPX1^−/−^	•No effect on age‐related neuromuscular ageing •increased RONS generation in skeletal muscle.	268
5LOX^−/−^	•No effect on surgical denervation‐induced muscle atrophy.	22
12/15LOX^−/−^	•Protected against surgical denervation‐induced muscle atrophy •prevented NADPH oxidase activity, protein ubiquitination and ubiquitin‐proteasome‐mediated proteolytic degradation.	22
TgSOD1^+/o^	•No effect on age‐related neuromuscular ageing •increased resistant to H_2_O_2_ cytotoxicity.	26
TgCAT^+/o^	•No effect on age‐related neuromuscular ageing •increased resistant to H_2_O_2_ cytotoxicity.	26
TgSOD1/CAT^+/o^	•No effect on age‐related neuromuscular ageing •increased resistant to H_2_O_2_ cytotoxicity.	26
SOD1^−/−^	•Accelerated neuromuscular ageing phenotype •loss of muscle fibres and CSA and increased number of centronucleated fibres •partial degeneration of NMJs, loss of innervation and motor function •impaired neurotransmitter release, reduced occupancy of the motor endplates by axons, fragmented postsynaptic endplates, terminal sprouting and axon thinning and irregular swelling •sciatic nerve demyelination and changes in neuron structure •reduced contractile force and grip strength •increased levels of oxidative damage and a constitutive activation of redox‐sensitive transcription factors •loss of mitochondrial integrity and function •elevated mitochondrial mediated apoptosis and caspase‐3 activity.	27, 29, 30, 33, 34, 274, 275, 276, 277, 278
mitoSOD1 SOD1^−/−^	•Prevented the biochemical and morphological defects in the SOD1^−/−^ model •rescued axon outgrowth and normalized mitochondrial density in primary motor neurons *in vitro* •prevented motor neuropathy and preserved NMJ integrity and grip strength.	23
mSOD1KO	•No effect on age‐related muscle atrophy •increased GTN skeletal muscle mass •increased degenerative‐regenerative phenotype and number of centronucleated fibres •reduced maximum isometric specific force.	34
SynTgSOD1^−/−^	•Prevented the neuromuscular ageing phenotype in the SOD1^−/−^ model •rescued age‐related muscle atrophy and muscle weakness •prevented degeneration of NMJ structure and function •no evidence of oxidative damage and adaptations in stress responses •no evidence of up‐regulated NFκB signalling.	29
nSOD1KO	•No effect on age‐related muscle atrophy of GTN, AT and EDL muscles •quadriceps and soleus showed a reduction in muscle mass •reduced maximum isometric specific force in GTN and EDL muscle •no effect on oxidative damage and adaptations in stress responses •altered NMJ morphology and increased expression of genes associated with denervation.	31

Knockout mice heterozygous for the MnSOD gene (SOD2^+/−^), mice with conditional knockout of MnSOD targeted to type IIB skeletal muscle fibres (TnIFastCreSod2^fl/fl^), mice overexpressing MnSOD (SOD2^Tg^), mice deficient in mitochondrial GPX4 (mGPX4‐KO), mice deficient in PRX3 (PRX3^−/−^), transgenic mice with targeted overexpression of the human CAT gene to mitochondria (mCAT^Tg^), transgenic mice with muscle specific over‐expression of rat nNOS (nNOS^Tg^), mice deficient in GPX1 (GPX1^−/−^), mice deficient in 5LOX (5LOX^−/−^), mice deficient in 12/15LOX (12/15LOX^−/−^), hemizygous transgenic mice that overexpress CuZnSOD (TgSOD1^+/o^), CAT (TgCAT^+/o^) and combined CuZnSOD and CAT (TgSOD1/CAT^+/o^), mice deficient in CuZnSOD (SOD1^−/−^), transgenic SOD1^−/−^ mice that exclusively expressed human SOD1 within the MIS (mitoSOD1,SOD1^−/−^), muscle‐specific CuZnSOD knockout mice (mSOD1KO), transgenic SOD1^−/−^ mice with neuron‐specific expression of CuZnSOD (SynTgSOD1^−/−^), neuron‐specific CuZnSOD knockout mice (nSOD1KO), gastrocnemius (GTN), anterior tibialis (AT), extensor digitorum longus (EDL), Akt–mammalian target of rapamycin (mTOR), neuromuscular junction (NMJ), mitochondrial intermembrane space (MIS).

### Genetic modification of mitochondrial redox systems to study the role of mitochondrial redox homeostasis in sarcopenia

Homozygotic mice lacking mitochondrial PRX3 isoform are viable with no signs of muscle atrophy, although this mouse model showed an increase in skeletal muscle mitochondrial ROS, altered mitochondrial morphology and decreased muscle fatigue resistance.^272^ These observations indicate that, although lack of PRX3 does not induce atrophy, it plays a crucial role in the contractile function of skeletal muscle by regulating the mitochondrial redox environment.^272^ Additional recent studies undertaken in the field of metabolomics have shown that, although homozygotic mice lacking either mitochondrial TRX2^247^ or TRX1^246^ have embryonic lethal phenotypes, specific deletion of TRX‐interacting protein in muscle specific knockout mice induces a reduction in exercise tolerance^279^ by maintaining the redox balance during exercise and preserving mitochondrial capacity to switch substrates during glucose deprivation.

Targeted overexpression of the human CAT gene to mitochondria in the mCAT^tg^ model has shown to protect against age‐induced deficits in muscle mitochondrial function, improve skeletal muscle respiratory function with age,^19^, ^242^ improve voluntary exercise and decrease the intracellular Ca^2+^ leak and the level of oxidized ryanodine receptor 1^32^. This occurs likely due to the attenuation of mito‐H_2_O_2_ which potentially reduces the reliance on antioxidant coupled NADPH‐driven reduction of oxidants in the mitochondria, thereby maintaining a higher availability of NADPH for exogenous antioxidant reduction.^280^

Increased oxidative damage and mitochondrial dysfunction have been proposed to contribute to the sarcopenic phenotype that occurs with ageing, and the findings in the preceding texts may suggest that scavenging of H_2_O_2_ specifically within skeletal muscle mitochondria may potentially rescue age‐related myofibre atrophy. However, studies have reported that the mCAT^tg^ model exhibits similar fibrosis levels and loss of muscle fibre size to age‐matched old wild‐type (WT) mice,^32^ indicating that reduced mitochondrial oxidative damage and improved mitochondrial function failed to rescue age‐associated muscle wasting. Similarly, heterozygous knockout of MnSOD^241^, ^266^, ^267^, ^268^, ^269^ and conditional knockout of MnSOD targeted to type IIB skeletal muscle fibres^25^, ^270^ showed no major effect on age‐related loss of muscle mass and structural changes. However, both these models showed mitochondrial functional deficits associated with elevated mitochondrial oxidative damage^25^, ^266^, ^269^ and reduced skeletal muscle aerobic capacity,^265^, ^270^ which support a role for MnSOD in regulating mitochondrial function and, subsequently, the aerobic capacity of skeletal muscle.

Moreover, recent studies using not only mice overexpressing the mitochondrial matrix SOD isoform (SOD2^Tg^)^24^ but also a double transgenic mouse model, SOD2^Tg^ combined with mCAT^Tg^,^35^ failed to preserve skeletal muscle mass with ageing 24 and showed no further improvements in insulin resistance in skeletal muscle of mice when fed on a high fat diet, indicating that increased mitochondrial superoxide scavenging does not improve muscle insulin action in mice fed on high fat diet alone or when coupled to increased H_2_O_2_ scavenging.^35^ Targeted disruption of the mitochondrial GPX4 isoform caused infertility in male mice, yet mitochondrial GPX4 isoform mouse model was fully viable, healthy in appearance, normal in behaviour and showed no difference in body size compared with WT siblings.^271^

In summary, data obtained from the available knockout and transgenic rodent studies, with a focus on mitochondrial redox systems, appear to support that the mitochondrial redox environment is critically important for embryonic development and plays an important role in regulating age‐related mitochondrial dysfunction, impaired mitophagy and aspects of skeletal muscle function. However, based on the available literature, there is limited evidence to suggest that the age‐related changes in mitochondrial redox potential contribute to the loss of muscle mass inherent with ageing. In support of this, recent ageing studies with use of mitochondria‐targeted antioxidants failed to provide evidence that defective mitochondrial redox signalling inherent with ageing is the key regulator of age‐related myofibre atrophy and weakness.^21^, ^39^

### Deletion of CuZnSOD in SOD1^−/−^ mice leads to accelerated neuromuscular ageing and functional deficits

**Figure 5:**
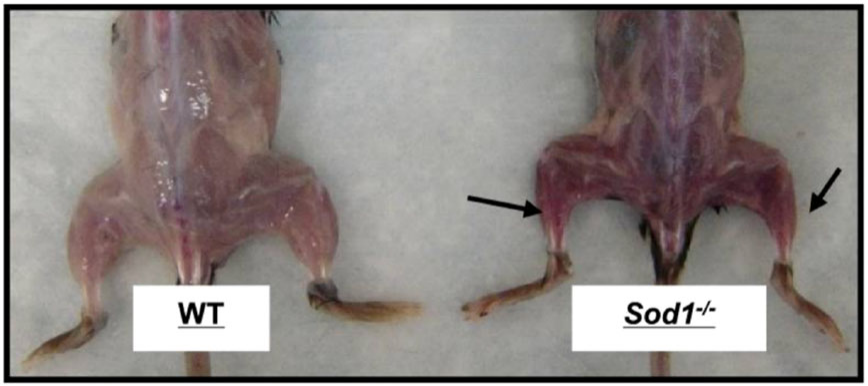
Gross morphology of skinned hindlimb muscles of SOD1^−/−^ and WT mice at 20 months of age. Redrawn from Jang *et al*. 2010.^276^

**Figure 6:**
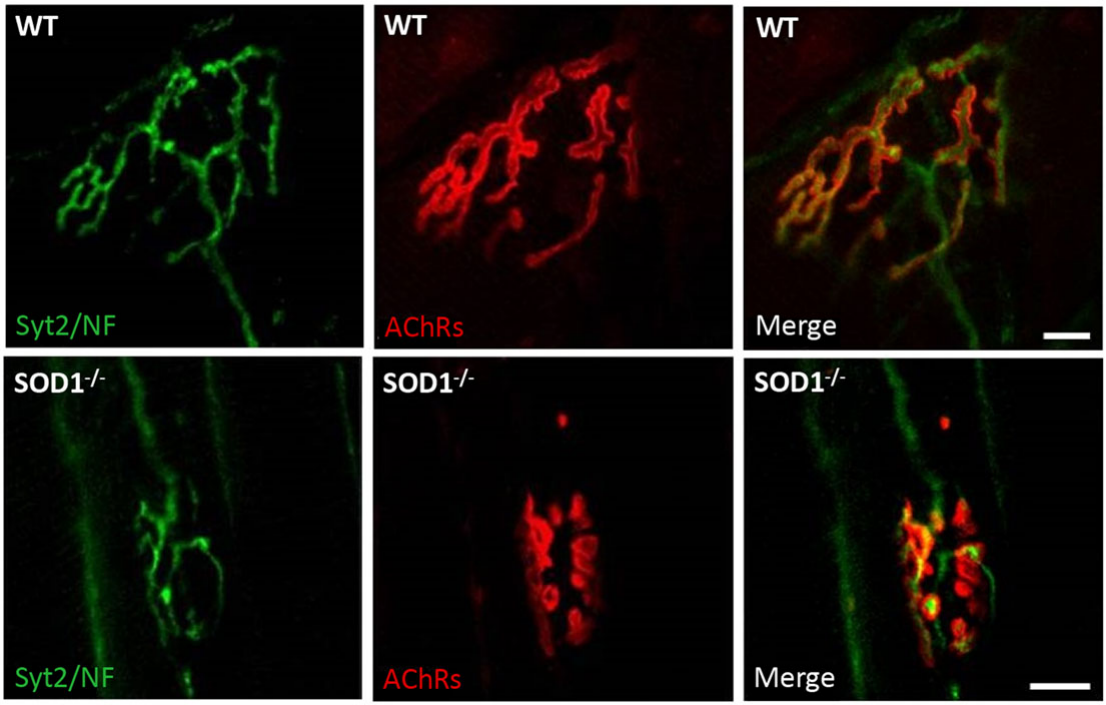
NMJ immunofluorescence images from AT muscle of SOD1^−/−^ and WT mice at 10 months of age. Left panels: morphology of presynaptic motor neurons stained with antibodies to synaptotagmin‐2 and neurofilaments (*green staining*). Middle panels: morphology of postsynaptic AChRs labelled with bungarotoxin (*red staining*). Right panels: merged images of presynaptic motor neurons and AChRs. Redrawn from Sakellariou *et al*.^29^ Original magnification: 60X (scale bar = 10 μm).

Reduced lifespan observed in SOD1^−/−^,^27^ PRX1^−/−^,^250^ PRX2^−/−^ 251 and TRX2^+/−^ 252 models is much more prominent in the SOD1^−/−^ rodent model,^281^ indicating that specific key RONS regulatory systems and redox signalling pathways are implicated in the processes of ageing. Moreover, although indistinguishable from WT mice at birth, by 5–8 months of age, SOD1^−/−^ mice show an accelerated neuromuscular ageing phenotype associated with myofibre atrophy (Figure 5), neurological impairments (Figure 6) and functional deficits.^275^ The features of the SOD1^−/−^ mouse model mimic those observed in 30 month old WT mice^27^, ^277^ and in older humans.^6^, ^277^ In addition, in common with old WT mice, skeletal muscle from SOD1^−/−^ rodents exhibits increased levels of oxidative damage^27^, ^29^, ^30^, ^31^, ^33^, ^34^, ^276^, ^277^ and a constitutive activation of redox‐sensitive transcription factors^33^; hence, it has been suggested that this knockout murine model represents a useful model for the study of chronic oxidative damage in the context of neuromuscular ageing in an effort to identify potential mechanisms and pathways that underlie sarcopenia in humans. It is noteworthy that hemizygous transgenic mouse models that overexpress CuZnSOD (TgSOD1^+/o^), CAT (TgCAT^+/o^) and combined (TgSOD1/CAT^+/o^) show no increase in lifespan and fail to rescue age‐related muscle wasting and functional deficits.^26^

The exacerbated neuromuscular ageing phenotype observed in the SOD1^−/−^ model implies that failure of redox homeostasis in specific subcellular compartments and/or tissues plays an important role in skeletal muscle ageing. As previously discussed, SOD1 is localized within the MIS and cytosol and catalyses the dismutation of superoxide to H_2_O_2_ and O_2_. Thus, the neuromuscular ageing phenotype observed in the SOD1^−/−^ model is likely associated with disrupted redox signalling within both the cytosolic and mitochondrial subcellular compartments. Elevated levels of oxidative damage and atrophy shown in skeletal muscle of SOD1^−/−^ are accompanied by increased mitochondrial generation of reactive species, impaired mitochondrial bioenergetic function and mitochondrial release of proapoptotic factors, which ultimately lead to apoptotic loss of myonuclei.^276^ These findings imply that the mitochondrial redox environment plays a central role in regulating skeletal muscle mitochondrial function. In support of this, the physiological and functional importance of maintaining redox homeostasis within the MIS was recently highlighted in a transgenic model that exclusively expressed SOD1 within the MIS (mitoSOD1,Sod1^−/−^) from SOD1^−/−^ mice.^23^ The transgenic approach used in the mitoSOD1,Sod1^−/−^ model prevented the morphological and biochemical defects associated with progressive motor axonopathy in skeletal muscle of the SOD1^−/−^ rodents,^23^ highlighting the importance of SOD1 redox regulatory enzyme expression in the MIS, and implicated oxidative damage initiated at mitochondrial sites in the pathogenesis of motor axon degeneration.

The accelerated muscle ageing phenotype observed in SOD1^−/−^ rodents may imply that excess levels of superoxide within the muscle cells are the underlying reactive species, responsible for the initiation and progression of muscle atrophy that occurs in this model. However, it is plausible to speculate that alternative RONS may play important roles in degeneration of neuromuscular integrity in the SOD1^−/−^ model, such as peroxynitrite or a change in NO bioavailability reacting with excess levels of superoxide. Studies using a combination of immunoblotting, high‐performance liquid chromatography and real‐time fluorescence microscopy methods have monitored specific intracellular RONS in single myofibres isolated from skeletal muscle of SOD1^−/−^ rodents. These studies carried out in resting and contracting skeletal muscle have demonstrated that genetic ablation of SOD1 does not induce the anticipated increase in cytosolic superoxide availability, but instead induced a substantial increase in peroxynitrite formation.^30^ These findings may provide important information of the RONS that are implicated in the processes of skeletal muscle ageing and highlight peroxynitrite formation as the important RONS mediator of the exacerbated neuromuscular ageing phenotype observed in the SOD1^−/−^ model. In support of these findings, ageing studies have provided evidence for increased peroxynitrite generation in skeletal muscle of old mice compared with adult mice, indicated by an increase in 3‐nitrotyrosine content of muscle proteins, suggesting that peroxynitrite might play an important role in the processes of neuromuscular ageing.^282^

Transgenic mice overexpressing nNOS isoform (nNOS^Tg^) also exhibit increased peroxynitrite formation in skeletal muscle.^30^ However, this model is not associated with changes in skeletal muscle morphology and function, in contrast to the SOD1^−/−^ murine model.^30^ A potential explanation might be due to the extent and the subcellular sites of peroxynitrite generation; nNOS is expressed along the sarcolemma of skeletal muscle fibres, whereas SOD1 is expressed in the cytosol and MIS. In support of this, skeletal muscle from SOD1^−/−^ mice (but not in nNOS^Tg^ mice) is associated with increased PRXV protein expression,^30^ an enzyme with a high peroxynitrite reductase activity,^144^, ^283^ predominantly localized in mitochondria.^147^ Hence, the increase in PRXV expression seen in muscle of SOD1^−/−^ mice supports a substantial increase in peroxynitrite in the mitochondrial organelles and highlights that specific RONS formed in specific subcellular compartments and/or tissues are implicated in the processes of neuromuscular ageing in the SOD1^−/−^ model. Additional research based on scavenging of the apparent age‐related increase in peroxynitrite generation is warranted to assess the role of peroxynitrite in the loss of neuromuscular integrity and function that occurs with the advance of age.

### CuZnSOD gene deletion targeted to skeletal muscle alone does not cause myofibre atrophy

Deciphering the key pathways and mechanisms underlying neuromuscular ageing has been challenging, in part because of the difficulty in unravelling the association between loss of motor units and loss of muscle mass, both of which occur with the advance of age.^284^ Motor nerves and muscles are well known to play a symbiotic role in maintenance of the neuromuscular system; specifically, the viability of motor neurons is recognized to be dependent upon continued exposure to neurotrophic factors released by myofibres.^94^ The prominent muscle ageing phenotype described in the SOD1^−/−^ model is associated with a number of neurological impairments, including gross alterations in NMJ morphology, reduced occupancy of the motor endplates by axons, fragmented postsynaptic endplates, terminal sprouting and axon thinning and irregular swelling, loss of motor function and contractility,^276^ impaired neurotransmitter release^278^ and loss of contractile force.^27^ In addition, induction of contraction by using direct muscle stimulation of muscle tissue, circumventing the NMJ, partially rescues the deficit in force, which indicates a loss of functional innervation in the SOD1^−/−^ model.^277^ Collectively, these observations may suggest that the age‐related deficits in muscle mass and force might be initiated by disrupted motor neuron redox signalling. However, whether the degenerative changes are initiated by altered redox homeostasis proximal and/or distal to the neuromuscular synapses remained inconclusive in these studies. In relation to this, reports have shown that age‐related changes in NMJ integrity^285^ and reduced muscle strength^286^ precede myofibre atrophy, highlighting the importance of the motor neuron system in neuromuscular ageing.

‘Conditional knockout models’, genetically engineered to lack or inactivate a gene of interest in a specific tissue or cell type, provide a valuable tool to examine the site‐specific importance of the function of that particular gene. Recent work has used Cre‐Lox targeted approaches to examine whether specific SOD1 gene deletion targeted to skeletal muscle (mSOD1KO) is sufficient to initiate the SOD1^−/−^‐associated sarcopenic phenotype.^34^ Surprisingly, mSOD1KO mice maintained muscle masses at or above those of WT control mice. Moreover, no detectable increases in global measures of oxidative damage or fibre RONS changes, no reduction in mitochondrial ATP production or adaptive stress responses were observed in muscle from mSOD1KO model.^34^ However, specific lack of SOD1 in skeletal muscle of mSOD1KO lead to a reduction of maximum isometric specific force and potentiated muscle regenerative pathways as shown by elevated Akt‐mTOR signalling and the presence of extensive central nucleation of muscle fibres.^34^ Collectively, these data reveal that, although SOD1 gene deletion targeted specifically to skeletal muscle induced specific functional deficits, loss of SOD1 protein expression restricted to skeletal muscle alone was not sufficient to cause muscle atrophy.^34^ These findings suggest that the altered muscle redox environment observed in SOD1^−/−^ is likely not the driving factor for the degeneration of NMJs and loss of muscle mass observed during ageing of the SOD1^−/−^ model.

### Neuron‐specific expression of CuZnSOD prevents the loss of muscle mass and function that occurs in SOD1^−/−^ mice

To unravel whether the muscle decline and weakness shown in the SOD1^−/−^ model is initiated by defective redox signalling within motor neurons, a recent study generated a transgenic SOD1^−/−^ model in which human SOD1 was expressed under the control of the synapsin 1 promoter (SynTgSOD1^−/−^), termed ‘nerve rescue’ mice.^29^ The experimental work undertaken in this study revealed that sciatic nerve SOD1 content in SynTgSOD1^−/−^ was 20% of control WT mice. Partial rescue of SOD1 expression in motor neurons of the nerve rescue mice reversed all aspects of the accelerated neuromuscular ageing phenotype observed in the SOD1^−/−^ model including the multiple biochemical and physiological changes associated with the exacerbated ageing phenotype.^29^ Increased oxidative damage and compensatory up‐regulation of redox regulatory enzymes, stress responses and adaptive signalling pathways observed in muscle from SOD1^−/−^ mice^30^, ^33^ were not present in the neuron‐specific transgenic SynTgSOD1^−/−^ model. Moreover, the accelerated degeneration in NMJ structure, including both presynaptic and postsynaptic NMJ features,^23^, ^276^ failure of neuromuscular transmission^29^, ^278^ and impaired *in situ* muscle‐force generation^34^, ^277^ that occur in the whole body SOD1^−/−^ model were completely rescued in the nerve rescue model.^29^

Expression of CuZnSOD in tissues that contain synapses, including brain, spinal cord, sensory and motor nerves in the SynTgSOD1^−/−^ model, excluded any role for other tissues and cell types, which may be anticipated to play an essential role in maintenance of NMJs (e.g. Schwann cells) or muscles (e.g. satellite cells). These findings highlight that failure of redox homeostasis in motor nerves alone is sufficient to generate an ageing phenotype in skeletal muscle and NMJs. This model provided a powerful approach to help elucidate the roles of tissue‐specific defects in redox status and highlights that redox homoeostasis in motor neurons plays a key role in neuromuscular ageing.

### Neuron‐specific reduction of CuZnSOD is not sufficient to initiate a full sarcopenia phenotype

The neuromuscular changes observed in the SOD1^−/−^ model of ageing have been further assessed in a model with targeted deletion of CuZnSOD specifically to neurons (nSOD1KO) by using Sod1‐floxed mice crossed to transgenic mice expressing Cre recombinase driven by the nestin promoter.^31^ The significant neuronal loss of CuZnSOD activity and protein expression in the nSOD1KO model was not sufficient to replicate the muscle atrophy and weakness observed in the SOD1^−/−^ model. Muscle mass from nSOD1KO mice was not altered in the gastrocnemius (GTN), anterior tibialis (AT) or extensor digitorum longus (EDL) muscles as opposed to the 30–45% reduction observed in adult SOD1^−/−^ 27, 277, 278 and 30–33 month old WT mice.^287^ Despite no change in mass, EDL and GTN showed a small but significant reduction in maximum isometric specific force (8–10% vs. ~30–40% in the SOD1^−/−^ model). Interestingly, quadriceps (~14%) and soleus (<10%) muscle of nSOD1KO mice showed a small but significant reduction in mass, associated with a trend for a reduction in myofibre size.^31^ Muscle mitochondrial reactive species generation and altered redox homeostasis and changes in protein expression on RONS regulatory enzymes were not increased in muscle from the nSOD1KO model. Moreover, although there was no evidence of denervation in the nSOD1KO model, NMJ morphology was altered (reduced endplate area) and the expression of genes associated with denervation acetylcholine receptor subunit alpha (AChRα), the transcription factor, Runx1 and GADD45α was increased, supporting a role for neuronal loss of CuZnSOD initiating alterations at the NMJ.^31^ The observed changes in NMJ structure/function were much less severe in the nSOD1KO compared with the SOD1^−/−^ model, with no evidence of NMJ fragmentation or denervation, which explains why the nSOD1KO model did not exhibit a similar neuromuscular ageing phenotype shown in the whole body SOD1^−/−^ model.

Collectively, based on the available data with use of conditional knockout and transgenic models, it appears that CuZnSOD deficits in either the motor neuron or muscle alone are not sufficient to initiate a full sarcopenic phenotype and that deficits in both tissues are required to recapitulate the loss of muscle and function observed in the SOD1^−/−^ model. The current evidence further suggests that alterations in NMJ morphology and function due to compromised redox homeostasis in motor neurons appear to be the prime event that potentiates muscle mitochondrial dysfunction and oxidative damage that triggers a retrograde response leading to further NMJ damage and dysfunction. Overall, these changes ultimately result in NMJ degeneration, failure of neuromuscular transmission, denervation, loss of muscle fibres, fibre atrophy and, eventually, sarcopenia.

### Genetic removal of 12/15‐lipoxygenase in 12/15‐LOX^−/−^ mice protects against denervation‐induced muscle atrophy

Denervation‐induced muscle atrophy, previously shown to not only stimulate the autophagy‐lysosome pathway^288^ but also up‐regulate several atrogenes that function as ubiquitin ligases to identify proteins for degradation by the proteasome,^289^, ^290^ has been further assessed in the 12/15LOX^−/−^ mouse model.^22^ Previous reports have shown that denervation‐induced muscle atrophy is associated with activation of cytosolic PLA_2_,^291^ an enzyme that regulates AA release from membrane phospholipids that act as a substrate for lipid metabolic pathways catalysed by LOXs, cyclooxygenase and cytochrome P450.^46^ Genetic ablation of 12/15‐LOX but not 5‐LOX showed protection against surgical denervation‐induced muscle atrophy,^22^ implying a selective role for the 12/15‐LOX pathway in neurogenic muscle atrophy. Removal of 12/15‐LOX (but not 5‐LOX) reduced NADPH oxidase activity, protein ubiquitination and ubiquitin‐proteasome‐mediated proteolytic degradation that were associated with neurogenic‐induced muscle atrophy.^22^ The findings from this study reveal a novel pathway for neurogenic muscle atrophy and suggest that 12/15‐LOX system may have important implications for neuromuscular diseases and neuromuscular deficits inherent with ageing.

Further murine models have been recently developed that resemble many key aspects of neurological impairment in muscle ageing and are also associated with muscle atrophy and contractile dysfunction. These models explore different mechanisms and have been described in recent reviews.^7^, ^292^

### Potential therapies to combat the age‐related deficits in skeletal muscle function

There is significant academic and commercial interest in the development of therapies, of both pharmacological and non‐pharmacological origin, to combat the loss of skeletal muscle mass and function, in the context of neuromuscular ageing and a wide range of myopathies.^293^ Physical activity is one of the most effective interventions known to delay the progression of several aspects of muscle ageing. Similar to rodent models,^41^, ^294^ human studies have shown that physical activity is beneficial in promoting survival of motor units,^11^ facilitating reinnervation of muscle fibres that become denervated secondary to impaired NMJ stability,^12^ in attenuating age‐related genotoxic stress^14^ and preserving redox regulated adaptive responses.^15^ However, there is also evidence that the plasticity of the NMJ to physical activity is attenuated with ageing and denervation may become exacerbated by exercise training in very old age.^7^ Specifically, aged rats subjected to long‐term exercise training exhibited greater muscle atrophy and myocyte oxidative damage compared with aged‐matched sedentary controls.^295^, ^296^ The potential for physical activity to induce adverse effects when initiated in old age has not been addressed in humans. Further studies (including also adjunct nutritional interventions) are needed to assess the plasticity of the neuromuscular human system in response to physical activity in advanced age, where the remodelled surviving motor units may be further compromised by increased muscle activation.

Antioxidants appear to be a logical intervention in combating the age‐related loss of muscle mass and function; however, to date, there have been no robust, longitudinal human studies carried out to address this. The use of broad‐spectrum antioxidants (i.e. vitamins C and E) initially seems a rather attractive proposition, as their mode of action is well established, their efficacy as antioxidants well described and are overall generally well tolerated. However, several studies, primarily from an exercise perspective, have investigated the impact of broad‐spectrum antioxidants on skeletal muscle, with the prevailing finding that RONS are in fact crucial components of the adaptive mechanisms within muscle—suggesting that antioxidant intervention in this context may have adverse effects.^297^ In addition, despite the causal role of aberrant redox homeostasis in the development of muscular dystrophy,^112^ early clinical trials using antioxidants such as vitamins B and E and penicillamine did not show any statistically significant clinical benefits.^298^ Similarly, use of pentoxifylline, a phosphodiesterase inhibitor with potent antioxidant and anti‐inflammatory activity, failed to provide any improvements on muscle strength and function in Duchenne muscular dystrophy patients,^299^ despite showing significant muscle strength restoration on mdx mice.^300^ The use of antioxidant therapies in muscular dystrophy has been described in a recent review.^293^

Calorie or dietary restriction has shown to promote survival in mammals and delay the onset of numerous age‐related phenotypes including sarcopenia.^301^, ^302^ At a biochemical level, calorie restriction interventions have shown to increase sirtuin 1 (a member of the sirtuin family linked to lifespan extension and enhanced mitochondrial biogenesis), the expression of peroxisome proliferator‐activated receptor α (PGC1α) (a master regulator of mitochondrial biogenesis and RONS defence system), thus reducing oxidative damage and preserving mitochondrial structural and functional integrity in metabolically active tissues of rodents and humans.^303^, ^304^, ^305^ A direct link among mitochondrial dysfunction, oxidative damage and neuromuscular innervation was recently established in which calorie restriction reversed or attenuated impaired muscle function, loss of innervation and the profound muscle atrophy exhibited in the SOD1^−/−^ mouse model.^287^ Specifically, dietary reduction improved mitochondrial function as evidenced by enhanced Ca^2+^ regulation, attenuated mitochondrial oxidative damage, reduced mitochondrial ROS production, increased MnSOD content and sirtuin 3 protein expression.^287^

Similarly, the use of branched‐chain amino acids (BCAAs) has also been shown to extend chronological life of rodents and promote muscle efficiency in mammals.^217^ Evidence has revealed that BCAA supplementation is coupled to sirtuin 1 expression, increased mitochondrial biogenesis and enhanced RONS protective pathways in middle‐aged mice, which ultimately improve the functional capacity of skeletal muscle including physical endurance and motor coordination.^217^ It is important to mention that the BCAA supplementation effects were attenuated in eNOS null mutant mice, indicating that BCAA‐mediated responses appear to be regulated by redox signalling pathways.^217^ Further longitudinal cohort investigations are needed to assess the potential effect of both calorie restriction and BCAA interventions on aspects of neuromuscular ageing in humans.

Recent development of novel antioxidant compounds, with a more specific mode of action (e.g. SS‐31 and MitoQ), has allowed researchers to assess specific mechanisms that may potentially alter the age‐related loss of muscle mass and function. Treatment of aged mice with SS‐31 peptide, a mitochondria‐targeted antioxidant, resulted in an overall decrease in markers of oxidative damage and improved specific aspects of skeletal muscle mitochondrial function, mitophagic potential and organelle integrity.^21^ However, SS‐31 drug treatment showed no impact on the features of sarcopenia including age‐related loss of myofibre CSA and muscle function.^21^ Another study in aged mice treated with SS‐31 reported improved mitochondrial energetics and increased resistance to fatigue.^306^ Collectively, these findings provide evidence that mitochondria‐derived ROS play a role in some of the aspects of musculoskeletal ageing.

The novel mitochondrial‐targeted antioxidant MitoQ has been a compound of significant interest that has undergone phase 1 and 2 clinical trials,^307^ to target the RONS‐mediated aspects of several pathologies (Parkinson's/multiple sclerosis) and for its potential impact on skeletal muscle. Although there is a large amount of evidence to suggest that MitoQ administration provides beneficial effects, recent studies in the field of muscle metabolism have shown that MitoQ was found to have no effect on exercise‐induced adaptations in muscle oxidative capacity in humans.^308^ Similarly, in the context of musculoskeletal ageing, MitoQ intervention in old mice failed to rescue the loss of muscle mass and function associated with ageing of skeletal muscle.^39^ Overall, targeted antioxidant compounds such as SS‐31 and MitoQ are clearly useful from a mechanistic perspective; however, the ability to translate these findings in a human context remains less clear. Additional research is warranted to facilitate our understanding on key areas of defective redox homeostasis and maintenance of neuromuscular integrity in humans. Longitudinal studies of ageing models and humans will help clarify the cause and effect relationships and thus identify relevant therapeutic targets to combat the age‐related deficits in skeletal muscle mass and function.

## Perspectives and future directions

Multiple theories have been proposed to explain the ageing process,^309^ but none has yet received wide acceptance. Nevertheless, the free radical theory of ageing seems to be the theory receiving the widest acceptance as a plausible explanation of the primary biochemical reactions at the basis of the ageing process, and during the last four decades, there has been an enormous increase of information on the effects of oxidants with age. There is considerable evidence in support of the free radical theory of ageing that comes from a series of studies with invertebrates. The recent technological advantages in the field of molecular genetics have enabled investigators to utilize genetic engineering techniques to alter specific redox genes or processes and examine whether redox homeostatic regulation plays a key role in mammalian ageing (including maximum lifespan, median lifespan and tissue/organ ageing).

Age‐related muscle atrophy and weakness, characterized by loss of lean muscle mass and reduced neuromuscular function, is a major contributor to frailty and loss of independence in the elderly, which has a major economic burden on the healthcare systems. Age‐dependent loss of muscle mass and strength is a multifactorial process involving a complex interaction of a variety of metabolic processes, and the primary biochemical and molecular mechanisms underlying this process have not been fully determined. Considerable evidence in both humans and various organisms has shown that skeletal muscle decline with advancing age is linked to an altered oxidative status of redox‐responsive proteins and increased oxidative modifications of macromolecules. Age‐related changes in redox homeostasis have been proposed to play‐a key role in sarcopenia as it underlies many age‐related human diseases including neurodegenerative disorders, neuromuscular diseases, skeletal muscle pathologies, ischemia‐reperfusion injury and diabetes. Over the past two decades, a series of knockout (whole body and tissue specific) and transgenic models have been generated to study whether the redox environment is linked to age‐related deficits in neuromuscular integrity and function. In the present review, we have outlined the genetic approaches that have been undertaken in rodent models and provide insights on the role of redox homeostasis in age‐related atrophy and weakness.

The majority of knockout and overexpressing mouse models failed to alter the neuromuscular ageing processes, which argue for a role of defective redox signalling in age‐related skeletal muscle loss and function implying that the free radical theory of ageing is not as simple and straight forward. Mice deficient in CuZnSOD show a reduction in lifespan and an accelerated neuromuscular ageing phenotype that resembles the biochemical and physiological changes observed in old WT mice and humans indicating that specific RONS regulatory enzymes and/or reactive species are implicated in the processes of muscle ageing. The striking alterations in NMJ integrity/function and loss of innervation observed in the SOD1^−/−^ mouse model highlight the implication of motor neuron integrity in myofibre atrophy and functional deficits. Compromised redox homeostasis of motor neurons as a potential mechanism of sarcopenia in CuZnSOD deficient mice has recently been underlined in a model with specific loss of CuZnSOD targeted to skeletal muscle alone but also in a ‘nerve rescue’ SOD1^−/−^ mouse model with neuron‐specific expression of CuZnSOD, suggesting that failure of redox homeostasis in motor neurons appears to be the prime event initiating sarcopenia during ageing. These studies have shed light on understanding (i) the redox mediated cross‐talk between skeletal muscle and motor neurons and (ii) the defective redox signalling events that underlie neuromuscular ageing.

To fully understand the key mechanisms through which redox homeostasis regulates age‐related neuromuscular integrity and function, further conditional knockout and transgenic models but also targeted interventions are warranted. Additional research will facilitate our understanding on key areas of defective redox homeostasis and maintenance of neuromuscular integrity. Collectively, this work highlights the important role of the redox environment in maintenance of neuromuscular integrity and function and suggests that defective redox signalling in motor neurons may contribute to age‐related deficits in skeletal muscle mass and function. Understanding fully the mechanisms through which the redox environment regulates neuromuscular integrity, muscle mass and function may uncover potential targets/sites for intervention for preventing sarcopenia in humans with the aim to improve the quality of life in the elderly.

## Conflict of interest

The authors declare that there is no conflict of interest.
